# Rare Primary Mitochondrial DNA Mutations and Probable Synergistic Variants in Leber’s Hereditary Optic Neuropathy

**DOI:** 10.1371/journal.pone.0042242

**Published:** 2012-08-03

**Authors:** Alessandro Achilli, Luisa Iommarini, Anna Olivieri, Maria Pala, Baharak Hooshiar Kashani, Pascal Reynier, Chiara La Morgia, Maria Lucia Valentino, Rocco Liguori, Fabio Pizza, Piero Barboni, Federico Sadun, Anna Maria De Negri, Massimo Zeviani, Helene Dollfus, Antoine Moulignier, Ghislaine Ducos, Christophe Orssaud, Dominique Bonneau, Vincent Procaccio, Beate Leo-Kottler, Sascha Fauser, Bernd Wissinger, Patrizia Amati-Bonneau, Antonio Torroni, Valerio Carelli

**Affiliations:** 1 Dipartimento di Biologia Cellulare e Ambientale, Università di Perugia, Perugia, Italy; 2 IRCCS Istituto delle Scienze Neurologiche di Bologna and Dipartimento di Scienze Neurologiche, Università di Bologna, Bologna, Italy; 3 Dipartimento di Biologia e Biotecnologie, Università di Pavia, Pavia, Italy; 4 UMR INSERM, U1083-CNRS6214, Angers, France; 5 University of Angers, School of Medicine, Angers, France; 6 University Hospital of Angers, Department of Biochemistry and Genetics, Angers, France; 7 Studio Oculistico D’Azeglio, Bologna, Italy; 8 Ospedale San Giovanni Evangelista, Tivoli, Italy; 9 Azienda San Camillo-Forlanini, Rome, Italy; 10 Unit of Molecular Neurogenetics, Pierfranco and Luisa Mariani Center for the Study of Children’s Mitochondrial Disorders, Foundation “C. Besta” Neurological Institute-IRCCS, Milan, Italy; 11 Centre de référence pour les Affections Rares en Génétique Ophtalmologique, Hôpitaux Universitaires de Strasbourg, Strasbourg, France; 12 Service de Neurologie, Fondation Ophtalmologique Adolphe de Rothschild, Paris, France; 13 Department of Ophthalmology, Saint Jean Languedoc Clinic, Toulouse, France; 14 Centre de Référence des Maladies Rares en Ophtalmologie, Consultationd ‘Ophtalmologie, HEGP, Assistance Publique – Hôpitaux de Paris, Paris, France; 15 Centre for Ophthalmology, University Clinics Tuebingen, Tubingen, Germany; 16 Department of Vitreo-Retinal Surgery, Center of Ophthalmology, University of Cologne, Cologne, Germany; 17 Molecular Genetics Laboratory, Institute for Ophthalmic Research, Centre for Ophthalmology, University Clinics Tuebingen, Tuebingen, Germany; Medical University of South Carolina, United States of America

## Abstract

**Background:**

Leber’s hereditary optic neuropathy (LHON) is a maternally inherited blinding disorder, which in over 90% of cases is due to one of three primary mitochondrial DNA (mtDNA) point mutations (m.11778G>A, m.3460G>A and m.14484T>C, respectively in *MT-ND4*, *MT-ND1* and *MT-ND6* genes). However, the spectrum of mtDNA mutations causing the remaining 10% of cases is only partially and often poorly defined.

**Methodology/Principal Findings:**

In order to improve such a list of pathological variants, we completely sequenced the mitochondrial genomes of suspected LHON patients from Italy, France and Germany, lacking the three primary common mutations. Phylogenetic and conservation analyses were performed. Sixteen mitochondrial genomes were found to harbor at least one of the following nine rare LHON pathogenic mutations in genes *MT-ND1* (m.3700G>A/p.A132T, m.3733G>A-C/p.E143K-Q, m.4171C>A/p.L289M), *MT-ND4L* (m.10663T>C/p.V65A) and *MT-ND6* (m.14459G>A/p.A72V, m.14495A>G/p.M64I, m.14482C>A/p.L60S, and m.14568C>T/p.G36S). Phylogenetic analyses revealed that these substitutions were due to independent events on different haplogroups, whereas interspecies comparisons showed that they affected conserved amino acid residues or domains in the ND subunit genes of complex I.

**Conclusions/Significance:**

Our findings indicate that these nine substitutions are all primary LHON mutations. Therefore, despite their relative low frequency, they should be routinely tested for in all LHON patients lacking the three common mutations. Moreover, our sequence analysis confirms the major role of haplogroups J1c and J2b (over 35% in our probands *versus* 6% in the general population of Western Europe) and other putative synergistic mtDNA variants in LHON expression.

## Introduction

Leber’s hereditary optic neuropathy (LHON), a blinding disorder characterized by subacute/acute loss of central vision that most frequently affects young males, is a maternally inherited condition associated with mitochondrial DNA (mtDNA) point mutations [1]–[3]. It is widely recognized that one of three common mutations, m.11778G>A, m.3460G>A and m.14484T>C respectively affecting the *MT-ND4*, *MT-ND1* and *MT-ND6* subunit genes of complex I, are present in over 90% of LHON patients. It is also now established that most of the so-called “secondary mutations”, which may enhance the pathogenic potential and penetrance of the m.11778G>A and m.14484T>C mutations, are linked to polymorphic nucleotide changes characterizing a rather frequent western Eurasian mtDNA haplogroup (haplogroup J) and its subclades [4]–[6].

Neurologists and ophthalmologists commonly encounter patients with a family history compatible with maternal inheritance, who fulfill the clinical criteria for LHON but lack the common mutations upon genetic testing. Complete mtDNA sequencing has shown in some of these cases the presence of different and rare nucleotide changes, most of these in the *MT-ND6* and *MT-ND*1 genes, which fit the accepted criteria for being pathogenic and these genes are now considered mutational hot spots for LHON [7], [8]. Mutations independently found in more than one unrelated case or family include those in *MT-ND6* (m.14482C>G/A, m.14568C>T, m.14495A>G), and in *MT-ND1* (m.4171C>A, m.3733G>A, and m.3635G>A) [7]–[21]. An additional mutation, validated in three families and in strict association with haplogroup J, is m.10663T>C in *MT-ND4L* gene [22]–[24]. A different set of putative pathogenic mutations has also been reported in single cases or families; these have been assigned a provisional status and await confirmation of their strict pathogenic association with LHON [1], [25], [26]. They include the variants m.3700G>A/*MT-ND1* [27], m.14498C>T/*MT-ND6* [12] and m.4640C>A/*MT-ND2* [18], the latter two are found in mutigenerational pedigrees with evidence of maternal inheritance. A different class of mutations, with a higher pathogenic potential leading to LHON plus MELAS (Mitochondrial Encephalomyopathy, Lactic Acidosis and Stroke-like episodes) or Leigh phenotypes, have also been described in *MT-ND6*, *MT-ND1*, *MT-ND3*, *MT-ND4* and, in particular, in the *MT-ND5* genes of complex I [26]. The prototype of this category is the mutation m.14459G>A/*MT-ND6* [28], originally found in a large pedigree affected by LHON, spastic dystonia with bilateral striatal necrosis or a combination of both [29], but subsequently identified in cases of fatal infantile Leigh syndrome [30]. Similarly, mutations in the *MT-ND5* gene are typically associated with a wide range of phenotypes, an example of which is the mutation m.13042G>A that is associated with both LHON and MELAS in the same maternal lineage, or may lead to overlapping MELAS/MERRF (Myoclonic Epilepsy, Ragged-Red Fibers) phenotypes or Leigh syndrome [31]–[33].

In the present study we analyzed 174 suspected LHON probands from unrelated families, lacking the three common mutations, and detected 16 mitochondrial genomes, harboring at least one rare pathogenic mutation. This study definitively establishes nine rare mtDNA point mutations as primary LHON mutations that should be routinely screened for if the three most common mutations are not identified.

## Results

**Figure 1:**
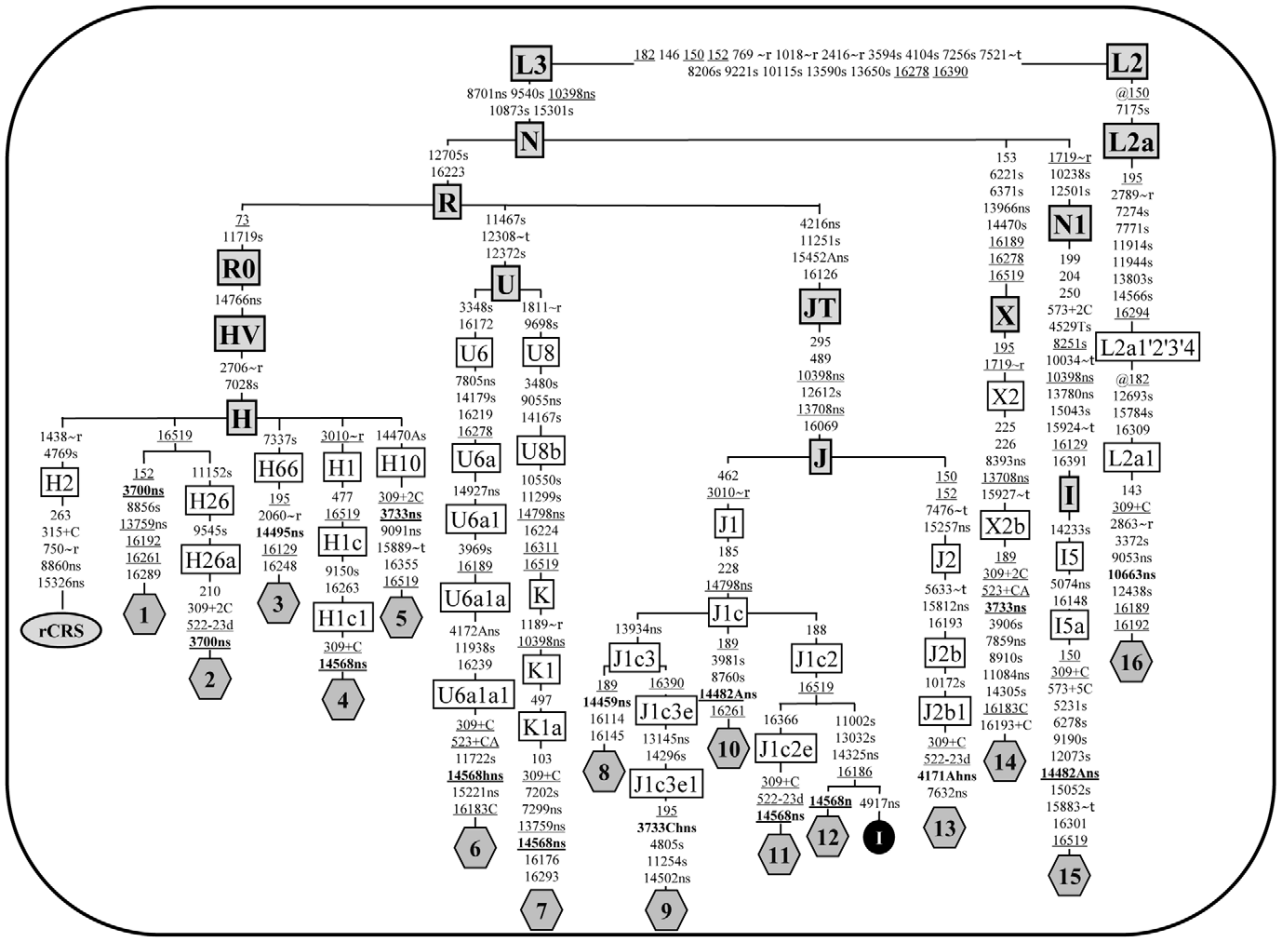
Phylogenetic tree of 16 complete mtDNA sequences from LHON patients. Rare LHON mutations are shown in bold. The position of rCRS [57] is indicated for reading off sequence motifs. Mutations are shown on the branches; they are transitions unless a base is explicitly indicated. The prefix @ designates reversions, while suffixes indicate: transversions (to A, G, C, or T), indels (+, d), gene locus (∼t, tRNA; ∼r, rRNA), synonymous or non-synonymous changes (s or ns) and heteroplasmies (h). Recurrent mutations are underlined. One mtDNA sequence (I; black circle) from a control subject (GenBank accession number FJ190383) was also included to illustrate that sequences 11 and 12 acquired independently the LHON mutation m.14568C>T. The haplogroup affiliation of each haplotype is based on mutational motifs and follows the most updated human phylogeny [35].

**Figure 2:**
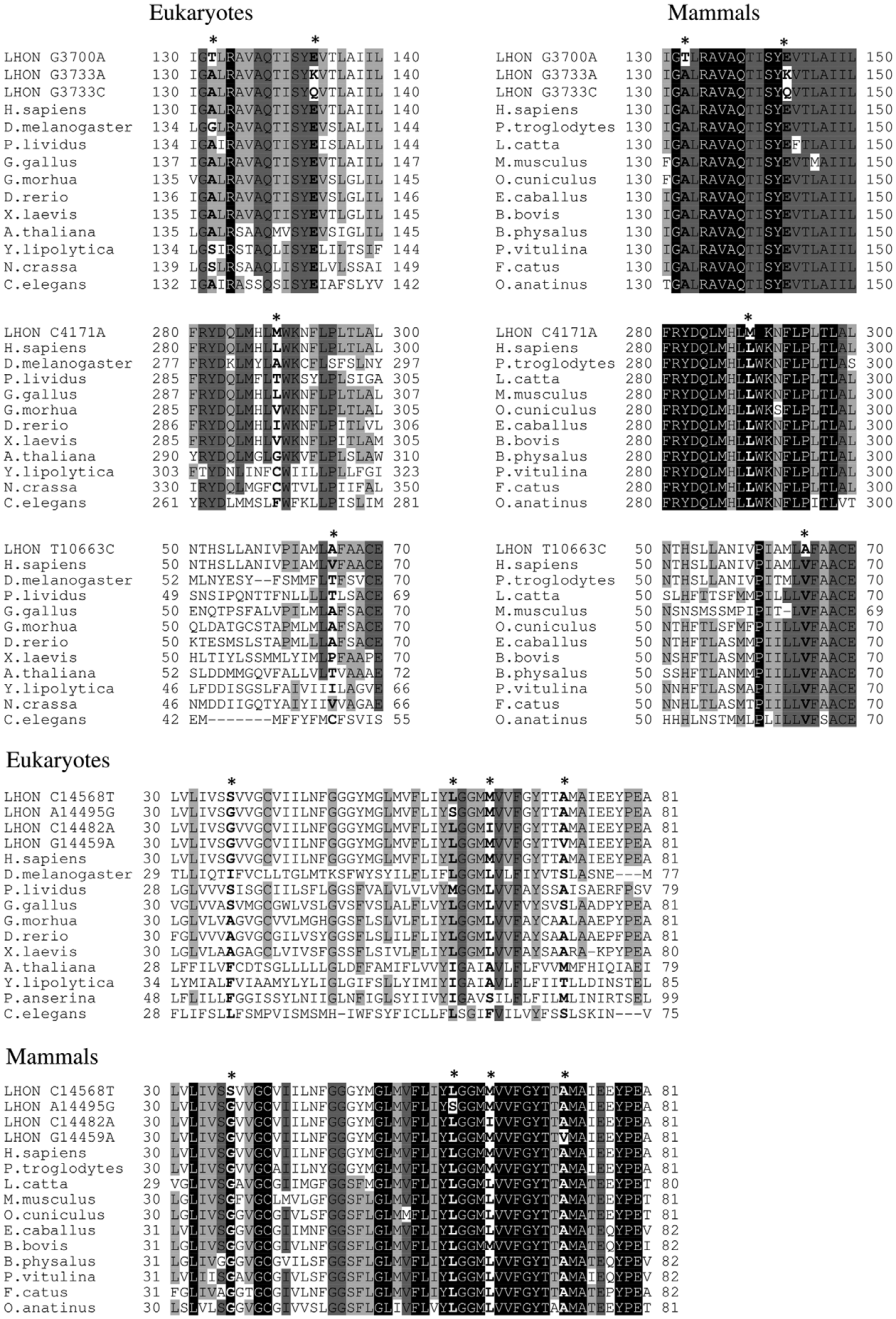
Sequence alignment and conservation analysis of ND1, ND4L and ND6 protein sequences. Alignments of protein sequences from eukaryotes and mammals, including *Homo sapiens,* are reported. The upper lines represent the human sequence with the amino acid changes induced by the corresponding LHON mutation. Mutated positions are in bold and indicated by an asterisk. Different shading corresponds to increasing conservation levels: amino acid conservation between 70% and 90% are highlighted in light grey, amino acid conservation between 90% and 99% are highlighted in dark grey, and invariant positions (100% conservation) are highlighted in black. Alignment gaps are indicated by a hyphen (-).

Among the three diagnostic centers involved in this study, sequencing of a total of 174 complete mitochondrial genomes was performed in cases highly suspected for LHON, but lacking the three common mutations (Table S1). Sixteen probands (Text S1) resulted positive for a rare pathogenic mutation and Figure 1 illustrates the phylogenetic relationships between these mitochondrial genomes. As expected the mtDNA sequence from French family 16, which is originally from Benin, belonged to a sub-Saharan African haplogroup (L2a1), whereas all other sequences clustered within a wide range of Western Eurasian haplogroups within macro-haplogroups N and R. Table 1 summarizes the main features of each mtDNA sequence. They were all different and each mtDNA was found to harbor at least one rare LHON mutation. Because several of the LHON mutations were found in more than one proband, the overall number of rare LHON mutations was nine (Tables 1 and 2). In all cases in which the same mutation was shared by more than one mtDNA, the haplogroup affiliation and the phylogenetic relationships between mtDNAs revealed that the sharing is not by descent but due to independent mutational events (Figure 1). All of them represent non-synonymous mutations that cause an amino acidic change in highly conserved position (>98% within mammals, see Table 2 and Figure 2), with the only notable exception of m.14482C>A/*MT-ND6* (p.M64I, conservation 58.14%).

**Table 1:** List of complete mtDNA sequences included in Figure 1.

FamilyID #^a^	Origin	Haplogroup^e^	Non-synonymous nucleotide changes^f^	GenBank accession number^g^	Reference
1	Italy	H	**3700**, 13759	JN415470	Present study
2	Germany	H26a	**3700**	JN415471	[27]
3	Italy	H66	**14495**	JN415472	Present study
4	Germany	H1c1	**14568**	JN415473	[12]
5	Italy	H10	**3733**, 9091	JN415474	[8]
6	Italy	U6a1a1	4172A, 7805, **14568**, 14766, 14927, 15221	EF064318.1	Present study
7	France^b^	K1a	7299, 9055, 10398, 13759, **14568**, 14766, 14798	JN415475	Present study
8	Italy	J1c3	4216, 10398, 13708, 13934, **14459**, 14766, 14798, 15452A	JN415476	Present study
9	Germany	J1c3e1	**3733C**, 4216, 10398, 13145, 13708, 13934, 14502, 14766, 14798, 15452A	JN415477	Present study
10	Italy	J1c	4216, 10398, 13708, **14482A**, 14766, 14798, 15452A	JN415478	[11]
11	France	J1c2e	4216, 10398, 13708, **14568**, 14766, 14798, 15452A	JN415479	Present study
12	Germany	J1c2	4216, 10398, 13708, 14325, **14568**, 14766, 14798, 15452A	JN415480	[14]
13	France	J2b1	**4171A**, 4216, 7632, 10398, 13708, 14766, 15257, 15452A, 15812	JN415481	Present study
14	Italy	X2b	**3733**, 7859, 8393, 11084, 13708, 13966, 14766	JN415482	[8]
15	Germany^c^	I5a	5074, 10398, 13780, **14482A**, 14766	JN415483	[10], [27]
16	France^d^	L2a1	8701, 9053, 10398, **10663**, 14766	JN415484	Present study

a Family ID numbers correspond to the numbers in Figure 1.

b This family is originally from Lebanon.

c This family is originally from Turkey.

d This family is originally from Benin.

e Haplogroup classification based on the most updated human mitochondrial phylogeny [35].

f Nucleotide positions and changes refer to rCRS [57]. The non-synonymous nucleotide changes at nps 8860 (8860G) and 15326 (15326G), present in all LHON samples, are not included because they are private mutations of the reference sequence. LHON mutations are in bold, while those with a possible synergistic effect are underlined.

g Only the entire mitochondrial sequence #6 was already published (Olivieri et al. [44]).

**Table 2:** Conservation analysis of rare primary LHON mutations.

Mutation Gene	Heteropl. Status	Amino AcidChange	Matches inGenBank^a^	EukaryotesCons. (%)	VertebratesCons. (%)	MammalsCons. (%)	PolyPhen2Predict.	SIFT Predict.	Family
m.3700G>A *MT-ND1*	–	p.A132T	0	79.22	93.52	98.73	Benign	Not Tolerated	1, 2
m.3733G>A *MT-ND1*	−/+ (ref. [8])	p.E143K	1 (FJ944094)	99.35	99.07	98.73	Possibly Damaging	Not Tolerated	5, 14
m.3733G>C *MT-ND1*	+ (78%)	p.E143Q	0	99.35	99.07	98.73	Probably Damaging	Not Tolerated	9
m.4171C>A *MT-ND1*	+ (80%)	p.L289M	0	60.39	84.26	100.00	Probably Damaging	Tolerated	13
m.10663T>C *MT-ND4L*	–	p.V65A	0	79.33	91.73	99.61	Possibly Damaging	Tolerated	16
m.14459G>A *MT-ND6*	–	p.A72V	1 (AY195748)	54.03	72.29	100.00	Probably Damaging	Not Tolerated	8
m.14482C>A *MT-ND6*	−/+ (ref. [11])	p.M64I	0	64.52	75.90	58.14	Probably Damaging	Not Tolerated	10, 15
m.14495A>G *MT-ND6*	+ (41%)	p.L60S	0	79.03	100.00	100.00	Probably Damaging	Not Tolerated	3
m.14568C>T *MT-ND6*	−/+ (60–90%)	p.G36S	2 (EF064318, EU545432)	34.68	51.81	100.00	Probably Damaging	Not Tolerated	4, 6, 7, 11, 12

a The GenBank database was searched on March 2^nd^ 2012 when a total of 10,304 mtDNA coding regions were deposited.

Seven of the nine mutations are already listed within the “Top 10” Primary LHON mutations in MITOMAP, while the m.3700G>A/*MT-ND1* transition is reported within the “other candidate LHON mutations found as single family or singleton cases”. The m.3733G>C/*MT-ND1* is not included in either of these lists. For the sake of clarity, the results for each LHON mutation are presented separately.

### m.3700G>A/*MT-ND1* (Families 1 and 2)

The mtDNAs from the probands of family 1 (Italy) (Figure S1) and the previously reported family 2 (Germany) [27] harbored the non-synonymous nucleotide change m.3700G>A. The phylogenetic relationship between the two probands’ sequences show that this mutation is not shared by descent, but it is due to two independent mutational events (Figure 1): the first radiates from the root of H, the most common haplogroup in Europe [34], whereas the second can be further classified into the H26a subclade [35]. This nucleotide change, never reported as a population polymorphism, causes the amino acid substitution p.A132T in the ND1 protein, which has been previously proposed as pathogenic for LHON in the German family [27]. The RFLP analysis of the m.3700G>A mutation showed that it was virtually homoplasmic in DNA from peripheral blood (data not shown). A DNA sample from urinary epithelium available only for individual II:1 of family 1 was also homoplasmic mutant. Both cases were apparently sporadic and no further DNA was available for analyses from maternal relatives. Thus, following the initial description of family 2, the pathogenic role of the rare m.3700G>A mutation is confirmed, with the detection of an Italian LHON patient (Family 1), as a separate mutational event.

Family 1 harbored also a second non-synonymous change m.13759G>A/*MT-ND5*. This nucleotide change is a polymorphic marker of the Asian haplogroups F1a’c’f and M7c1d [35], and it has been also sporadically observed in mtDNAs belonging to other haplogroups.

### m.3733G>A-C/*MT-ND1* (Families 5, 9 and 14)

The m.3733G>A mutation was detected in two previously described Italian families (Families 1 and 2 in Valentino et al. [8], here renamed families 5 and 14, respectively) on haplogroups H10 and X2b, thus indicating that it resulted from two distinct mutational events (Figure 1). The mutation m.3733G>A causes the amino acid substitution p.E143K and in both families was homoplasmic in the affected individuals, whereas many unaffected maternal relatives were heteroplasmic [8].

The mtDNA from family 5 revealed the presence of only the other non-synonymous change m.9091A>G/*MT-ATP6*, which has been found in several healthy subjects belonging to haplogroup H10 [35], thus a role in LHON pathogenicity is very unlikely.

The mtDNA sequence from family 14 was instead characterized by numerous non-synonymous changes (see Table 1). As shown in Figure 1, most of these (m.8393C>T, m.13708G>A and m.13966A>G) are ancient polymorphisms of haplogroup X2b. The remaining two substitutions (m.7859G>A and m.11084A>G) are phylogenetically more recent. The m.7859G>A nucleotide change was previously reported in a pediatric case of mitochondrial encephalopathy (patient 2 in Uusimaa *et al.* [36]), but no clear pathogenicity was assigned because it was homoplasmic. Moreover, it has also been found in different normal individuals associated with at least two different Asian haplogroups (Y2 and M4) [35]. Similarly, the m.11084A>G substitution has also been initially reported as pathogenic for MELAS in an Australian patient [37], but subsequently identified as a polymorphism of the Asian haplogroups A4e1 and M2a1a1a [35]. Thus, most probably these changes are polymorphic variants, but their potential synergistic role is further discussed based on their conservation pattern.

**Figure 3:**
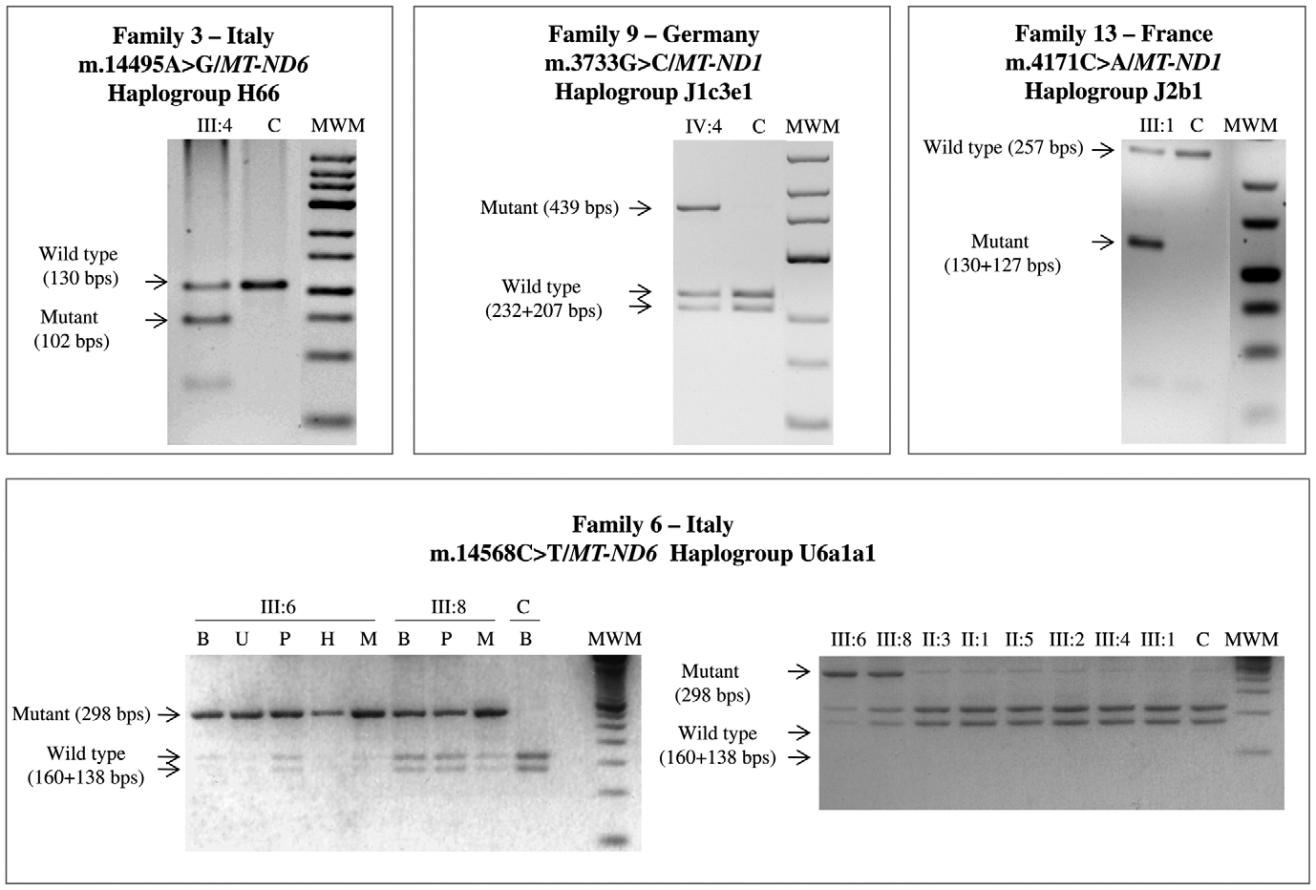
RFLP evaluation of heteroplasmy. Electrophoresis on Metaphor gel of the restriction fragments is shown. Wild type and mutant fragments are indicated with the corresponding size, expressed as base pairs (bps). Non affected negative control individual is indicated as C. DNA samples were extracted from whole blood, except when indicated: whole blood is indicated as B, urinary epithelium is indicated as U, platelet fraction is indicated as P, hairs are indicated as H and skeletal muscle is indicated as M. Molecular weight marker is indicated as MWM.

In the course of this study we also found a family from Germany (Family 9 in Figure S1) harboring a heteroplasmic (see Figure 3) variant at the very same position but with a different nucleotide change, m.3733G>C, leading to a different amino acid substitution (p.E143Q). Its mtDNA belongs to sub-haplogroup J1c3e1, a clade characterized by several non-synonymous variants affecting *MT-ND1-6* and *MT-CYB* genes (Table 1, Figure 1). Beside those changes established as markers of J1c3e1, we also note the presence of the change m.14502T>C/*MT-ND6*. This variant has been previously reported as the only pathogenic change in three unrelated sporadic LHON cases from China, in one Chinese LHON pedigree characterized by complete penetrance in combination with the m.14484T>C primary LHON mutation, and in four additional Chinese unrelated families carrying the m.11778G>A mutation [38]–[40]. The latter two scenarios parallel the one that we observed in the mtDNA sequence of family 9, where the m.14502T>C mutation is in association with the heteroplasmic m.3733G>C mutation. In this context, the m.14502T>C change might worsen the complex I deficiency caused by the 3733G>C transversion.

### m.4171C>A/*MT-ND1* (Family 13)

The complete sequencing of mtDNA from the proband of the small French family 13 (Figure S1), with two maternally related affected males revealed the heteroplasmic m.4171C>A mutation (Figure 3), which was previously proposed as pathogenic in two Korean families [15] and further found in a sporadic case and one family from China [16], [17]. Both Korean mtDNAs harbored apparently identical sequences belonging to haplogroup A, thus raising the possibility of a single ancestral mutational event. However, in one family the proband was apparently a sporadic case and his mother was heteroplasmic, supporting the alternative scenario of two independent mutational events. The Chinese cases were unrelated to the Korean, and one family (haplogroup N9a1) displayed an almost complete penetrance [17]. In all these cases further changes affecting *MT-ND1-6* and *MT-CYB* genes were thought to be putatively synergistic with the m.4171C>A mutation. The mtDNA of our proband belongs to haplogroup J2b1 (Figure 1) and this is the first report of the m.4171C>A mutation associated with LHON on a non-Asian background.

The addition of our case, obviously unrelated, confirms the pathogenic role of the m.4171C>A mutation and its association with haplogroup J2b (presenting numerous non-synonymous polymorphisms) and further suggests the need for synergistic variants to increase its pathogenicity. Moreover, this mtDNA also harbored another novel variant, m.7632T>C/*MT-COII*, whose synergistic role cannot be excluded (Table 1).

### m.10663T>C/*MT-ND4L* (Family 16)

The complete sequencing of mtDNA from the proband of this sporadic French case of African descent (Benin) revealed a haplogroup L2a1 background (Figure 1 and Figure S1) carrying the m.10663T>C mutation. This mutation was previously reported in five pedigrees all belonging to different haplogroup J sub-clades [23], [24], thus indicating multiple independent mutational events. Our case is the first in a non-J mtDNA background and harbored also a combination of non-synonymous polymorphisms m.8701A>G/*MT-ATP6*, m.9053G>A/*MT-ATP6* and m.10398A>G/*MT-ND3*, for which a synergistic role, similar to the haplogroup J polymorphisms, may be considered.

### m.14459G>A/*MT-ND6* (Family 8)

The LHON proband from this Italian family carried a virtually homoplasmic m.14459G>A mutation (Table 1 and 2, Figure S1). The pathogenicity of this mutation is well established and notorious for a variable clinical expression, ranging from LHON, dystonia or both, and Leigh syndrome [28], [30], [41], [42]. Here, two maternal relatives of the proband, reported as wheelchair bound and suffering of “paralysis”, were possibly affected with spastic dystonia. The mtDNA of the family here reported belongs to haplogroup J1c3, thus reinforcing the previously discussed synergistic role of this clade.

### m.14482C>A/*MT-ND6* (Families 10 and 15)

Both families, one Italian and one German of Turkish ancestry, were previously reported to carry the m.14482C>A mutation [11], [27], but the complete sequence analysis was not available. The Italian sequence belongs to haplogroup J1c and harbors all the non-synonymous variants characterizing this haplogroup, whereas the German family is a member of haplogroup I5a, which is also characterized by three non-synonymous variants m.5074T>C/*MT-ND2*, m.10398A>G/*MT-ND3* and m.13780A>G/*MT-ND5*. A different nucleotide change affecting the same position m.14482C>G/*MT-ND6* has been previously reported [9] in an unrelated family of Turkish ancestry, which noticeably belonged to the haplogroup I. It is conceivable that haplogroup I potentially plays a role similar to J. Furthermore, a third patient carrying the m.14482C>A mutation has been reported by screening a large cohort of patients with optic atrophy from France [43]. In summary, mutations at position 14482, although affecting a poorly conserved amino acid (Table 2) that is also changed by the well-established LHON primary mutation m.14484T>C, must be considered pathogenic.

### m.14495A>G/*MT-ND6* (Family 3)

The proband from the Italian family 3 carried the heteroplasmic m.14495A>G mutation (see Table 2, Figure 3 and Figure S1), which represents the third unrelated occurrence of this mutation in association with LHON [7]. All the cases, including our (sub-haplogroup H66), belong to haplogroup H and the mutational events were possibly recent, as suggested by their heteroplasmic status.

### m.14568C>T/*MT-ND6* (Families 4, 6, 7, 11 and 12)

This previously reported transition [12]–[14] was found in five different mtDNAs, whose phylogenetic analysis indicates independent mutational events (Figure 1).

In the mtDNA from family 6 (haplogroup U6a1a1, Figure S1) the m.14568C>T mutation was found heteroplasmic in combination with five non-synonymous nucleotide changes (see Table 1 and Olivieri *et al.* [44]). They are all known polymorphic variants, with the m.4172T>A mutation defining this specific subclade. This polymorphism induces the p.L289Q change by affecting the same codon of the previously discussed m.4171C>A pathogenic mutation. In addition, the m.14568C>T mutation was heteroplasmic in multiple tissues from the proband, being approximately 90% in skeletal muscle and urinary sediment, but 60% in blood leukocyte and platelet fractions. Similar results were obtained by analyzing multiple tissues from the proband’s brother, whereas all the remaining maternally related individuals available for analysis harbored a virtually homoplasmic wild type mtDNA except for the proband’s mother and maternal aunt who had traces of mutant mtDNA (Figure 3). The RFLP analysis of the m.4172T>A variant revealed that it was homoplasmic in all maternal relatives and all tissues investigated (data not shown).

The same pathogenic mutation m.14568C>T was also found in two unrelated French families, which belonged to haplogroups K1a (family 7) and J1c2e (family 11), respectively. In both cases the mutation was homoplasmic in the probands. Besides the haplogroup J-related non-synonymous changes of mtDNA from family 11, the mtDNA from family 7 harbored a collection of non-synonymous variants (Table 1).

Finally, we also re-examined by complete mtDNA sequencing the two originally reported German cases carrying the m.14568C>T mutation (families 4 and 12) [12], [14]. The proband from family 4 belonged to haplogroup H1c1 with no further non-synonymous changes besides the pathogenic mutation, whereas the proband from family 12 clustered within haplogroup J1c2, thus harbored all non-synonymous changes characterizing this haplogroup [35]. Overall, two of the five families carrying the m.14568C>T mutation were associated with haplogroup J1c, and one further family had the interesting association with a variant affecting the same amino acid changed by the LHON pathogenic m.4171C>A mutation, again emphasizing the importance of the mtDNA background sequence variation.

### Interspecies Conservation Analysis of Pathogenic Mutations and Non-synonymous Variants

The mutation m.3700G>A/*MT-ND1* causes the substitution of the non-polar p.A132 with a polar T and affects a highly conserved position (Table 2). Similarly, the mutations m.3733G>A/*MT-ND1* and m.3733G>C/*MT-ND1* induce the amino acid substitutions p.E143K or p.E143Q, respectively. The position affected is extremely conserved (Table 2) and both mutations cause a change in the chemical properties from the acidic glutamic acid, which is negatively charged, to either the positively charged, basic lysine or the uncharged, polar glutamine. Remarkably, these three mutations (m.3700G>A, m.3733G>A and m.3733G>C) affect the same highly conserved region of the ND1 protein (Figure 2), suggesting an important role of this conserved domain in complex I function or structure. The mutation m.4171C>A/*MT-ND1* also affects the highly conserved position p.L289 (Table 2), which is invariant in mammals, and leads to the substitution with methionine. Interestingly, the polymorphism m.4172T>A/*MT-ND1*, found in family 6 mtDNA and considered a polymorphic marker of haplogroup U6a2, affects the same p.L289, causing the replacement of the non-polar leucine with the negatively charged glutamic acid, suggesting a probable synergistic role of this polymorphism in LHON pathogenesis co-occurring with the primary mutation m.14568C>T/*MT-ND6*.

The m.10663T>C/*MT-ND4L* mutation induces the conservative amino acid substitution valine to alanine at position 65; the residue is moderately conserved along eukaryotes but becomes highly conserved in vertebrates and mammals (Table 2).

Four of the five mutations in the *MT-ND6* gene are concentrated in a hyper-conserved region of the protein and mostly affect highly conserved positions. The m.14459G>A/*MT-ND6* mutation causes a conservative p.A72V replacement. The p.A72 is highly conserved along vertebrates and becomes invariant in mammals, underlying a major functional role of this amino acid in the ND6 subunit. Similar features characterize the amino acid substitution generated by the mutation m.14495A>G/*MT-ND6*. This mutation causes the replacement of the highly conserved non-polar p.L60, which is invariant in vertebrates and mammals (Table 2), with a polar serine. Within this highly conserved amino acid stretch of ND6, a remarkable exception is the residue p.M64, affected by the mutations m.14482C>A-G. In fact, this mutation induces a conservative change (p.M64I) and hits a position with a low degree of conservation (see Table 2 and Figure 2) which is, however, contiguous to invariant stretches of amino acid positions characterizing this highly conserved domain of the *MT-ND6* gene in mammals (Figure 2). Finally, the m.14568C>T/*MT-ND6* mutation affects the amino acid residue p.G36 that is moderately conserved along eukaryotes and vertebrates, but becomes invariant in mammals, again suggesting the acquisition of a particular role of this position during evolution. Two different predictive tools (PolyPhen2 and SIFT) were also used to evaluate the pathogenic potential of the nine novel rare mtDNA variants. All mutations were classified as “possibly/probably damaging” or “not tolerated” by at least one software, and for 67% of these (six out of nine, Table 2), both algorithms highlighted the possible pathogenic effect.

Considering the 28 non-synonymous polymorphic variants identified in the 16 completely sequenced mtDNAs (see Figure 1), 14 were over the threshold of 70% conservation in mammals (Table S2). As already noted, the m.4172T>A/*MT-ND1* polymorphism, a marker of haplogroup U6a2, stands out by affecting the same invariant p.L289 (100% conservation) as the LHON primary mutation m.4171C>A/*MT-ND1*. A further group of polymorphisms with very high degree of conservation in mammals (90%–100%) included five variants: m.7632T>C is a private mutation of a J2b1 sample; m.13145G>A and m.15257G>A are markers of J subclades; the remaining two (m.9055G>A and m.14927A>G) define two different U branches (U8b and U6a1, respectively). Other seven polymorphisms had a conservation pattern between 70% and 90%. Finally, we also applied the two *in-silico* predictors (PolyPhen2 and SIFT) to further assess the functional impact of these polymorphic changes on mtDNA encoded proteins. Nine amino acid substitutions were independently predicted as damaging or not tolerated by these tools (Table S2). As expected, the polymorphism m.4172T>A/*MT-ND1* is the only one that is predicted to have a functional impact by both algorithms, because it hits the same p.Leu298 amino acid of the LHON primary mutation m.4171C>A/*MT-ND1*, which is invariant in mammals.

To further characterize the possible functional role of the non-conserved polymorphic variants, we extended the conservation analysis on the protein region surrounding these polymorphisms. Eight out of 15 had an invariant amino acid within four contiguous amino acidic positions (+4/−4 aa, Table S3) and four of these hit a protein region with a local conservation higher or equal to the global conservation of the protein subunit, suggesting that the protein regions containing these variants may represent functional domains. Remarkably, two ancient variants defining haplogroups J (m.13708G>A) and X (m.13708G>A and m.8393C>T) were selected by both analyses, supporting their possible functional role. In particular, the m.13708G>A/*MT-ND5* variant has been repeatedly associated with LHON as a secondary mutation.

## Discussion

Sequencing of the mitochondrial genomes from 16 sporadic or familial LHON cases, selected by using stringent clinical criteria and the strict maternal segregation of the phenotype, revealed nine rare point mutations with a number of features routinely employed to validate a pathogenic mtDNA mutation: (i) they arose multiple times on different mtDNA backgrounds (Figure 1); (ii) they affect conserved amino acid positions (Figure 2); (iii) they are virtually absent from population surveys and public datasets (see Table 2); (iv) they are strictly associated with LHON; (v) they are, in some instances, heteroplasmic along the maternal line indicating a recent *de novo* event (Figure 3). Overall, the rare mutations m.14568C>T/*MT-ND6* (6 cases), m.10663T>C/*MT-ND4L* (6 cases), and m.4171C>A/*MT-ND1* (5 cases) were found more frequently, whereas others were found less frequently such as m.14482C>A-G/*MT-ND6* (4 cases), m.14495A>G/*MT-ND6* (3 cases), m.3733G>A-C/*MT-ND1* (3 cases) and m.3700G>A/*MT-ND1* (2 cases). Concerning the m.14459G>A mutation, this has been clearly established as pathogenic in multiple pedigrees expressing a phenotype of variable severity, ranging from LHON to spastic dystonia or Leigh syndrome. Thus, we may consider this mutation as belonging to a different category of LHON “plus” mutations.

Multiple occurrences on different mtDNAs haplogroup of putative primary LHON mutations are crucial to assign a pathogenic role. In most cases the mutations listed above were homoplasmic along multiple generations of single maternal lineages. However, in a few families, we have been able to document mutations in heteroplasmic state in close maternal relatives of the probands (Figure 3), thus showing a recent occurrence of the mutational event, which has been frequently suggested as additional evidence of pathogenicity. In two families, we observed the co-occurrence of two nucleotide changes with the primary mutation and in both cases they may play a relevant role for the pathogenic expression of LHON. In one case, family 9, one mutation (the heteroplasmic change m.3733G>C) could be considered pathogenic, while the other (m.14502T>C) could modulate its penetrance. In a few other cases previously reported, the co-occurrence of two pathogenic mutations was noted, and in most instances one of these was heteroplasmic [45]–[47].

Important criteria used to validate a pathogenic mutation are the degree of interspecies conservation of the affected amino acid position and the physical/chemical characteristics of the amino acid change. All primary mutations affected highly conserved or invariant amino acid positions or less conserved amino acid positions within highly conserved protein domains, in particular when restricting the analyses to mammals. Most primary mutations induced non-conservative amino acid changes, frequently introducing drastic modifications in the specific residue. Concerning the ND1 subunit, all confirmed LHON mutations affect highly conserved extra-membrane loops, whereas most of the confirmed LHON mutations in ND6 affect the third trans-membrane helix, the most conserved in ND6 protein. These findings firmly establish that both *MT-ND6* and *MT-ND1* genes are hot spots for LHON mutations, as previously suggested [7], [8]. The functional relevance of these mutations is not assessed in the present study, and formal demonstration of the biochemical consequences is under way by transferring the mutant mtDNAs to cybrid cell lines [48]. However, considering the recent breakthrough in understanding complex I function due to its crystal structure elucidation [49], [50], we may anticipate that *MT-ND1* mutations will affect more consistently the electron transfer to the quinone substrate and complex I redox activity, whereas *MT-ND6* mutations may be more relevant for the “piston-driven” proton translocation and the energy conserving function [51]–[53].

The complete sequence data for the 16 mtDNAs here investigated also highlight the role played by certain haplogroup-specific non-synonymous variants, as well as the frequent co-occurrence of private sets of putative synergistic non-synonymous changes or double mutants (Table 1), all potentially relevant in modulating penetrance and severity of the primary LHON mutations. The high occurrence of haplogroups J1c and J2b (over 35%) in our limited set of patients is 5.6 fold that observed in the general population of Western Europe (6.2% in total; 5.3% in France, 6.6% in Germany and 6.2% in Italy) [54]. This finding further supports the role of these two mtDNA backgrounds in increasing LHON penetrance. A similar effect may be played also by haplogroup I, as suggested by the association with the m.14482C>A-G mutation [9], [27]. An apparent paradox is represented by the m.4171C>A LHON primary mutation and the m4172T>A polymorphic variant, both affecting the same amino acid position in ND1, but inducing different amino acid changes, p.L289M and p.L289Q respectively. The less conservative p.L289Q change is apparently tolerated as a polymorphism in the haplogroup U6a [35], whereas the p.L289M has been established as an LHON primary rare mutation [15–17 and present study]. Thus, the mtDNA background is remarkably important in determining the role of amino acid changes affecting the same position in a different mtDNA context. Such a borderline situation applies also to the m.14484T>C and m.14482C>A-G mutations in *MT-ND6*. In fact, they affect one of the less conserved amino acid positions among LHON primary mutations and represent, by far, the best example of the need for a tight association with specific haplogroups (J and I) to express their pathogenic potential. Compatibly, the m.14484T>C mutation has been identified in a few instances as a non-synonymous variant in population surveys, apparently without expressing any pathological phenotype (Antonio Torroni, unpublished observation and ref. [35]). Furthermore, it must be recalled that this mutation was judged as a non-pathogenic variant when it was first observed in LHON pedigrees [55]. Thus, we may predict that the m.4172T>A change behaves as a polymorphic variant in the context of haplogroup U6a, and may exert a pathogenic role if found in a different mtDNA background. Remarkably, both affected individuals in family 6 of the present study were heteroplasmic with variable mutant loads of the heteroplasmic m.14568C>T primary mutation co-occurring with the homoplasmic m.4172T>A change, suggesting a possible synergistic role for the latter. The blurry scenario revealed by these findings complicates the set of rules necessary to clearly assign a pathogenic role to a non-synonymous homoplasmic change in mtDNA and emphasizes the importance of sequencing the entire mtDNA in every LHON case. The assignment of a pathogenic role for an mtDNA variant remains a complex issue, as recently documented for the m.3394T>C mutation, which may behave as a weak pathogenic mutation for LHON or an adaptative polymorphic variant depending on the mtDNA background [56].

Based on this study we propose a revised diagnostic flow chart for LHON, which may include the following steps: (a) standard screening for the three common primary mutations; (b) if negative, sequence analysis the *MT-ND6* and *MT-ND1* genes; (c) if negative, complete mtDNA sequencing. The latter may be an option, when possible, to exhaustively study any case of LHON.

In conclusion, the nine mutations validated here should now be included as confirmed primary LHON mutations in public databases such as MITOMAP. These mutations are rare, but occurred multiple times in maternal lineages, and probably account for an important fraction of the about 10% of LHON patients who are negative for the three common mutations.

## Methods

### Ethics Statement

All experimental procedures and written informed consent, obtained from all donors, were reviewed and approved by the following Ethics Committee: Comitato etico indipendente dell’Azienda Ospedaliero-Universitaria di Bologna, Policlinico S.Orsola-Malpighi, Prot. n. 789/2007.

### Mitochondrial DNA Complete Sequencing and Phylogenetic Analyses

DNAs were extracted from venous blood. Sequencing of entire mtDNAs and phylogeny construction was performed as previously described [34]. All the mutations are relative to the revised Cambridge Reference Sequence (rCRS) [57]. The complete mtDNA sequences of the 16 case reports have been deposited in GenBank (Table 1).

### Evaluation of Heteroplasmy for Rare LHON Mutations

Heteroplasmy/homoplasmy status of the rare LHON mutations was evaluated by RFLP analysis of PCR amplified mtDNA fragments encompassing the position of the mutant nucleotide. The list of the primers and restriction enzymes used for each mutation are listed in Table S4. Fragments were separated by electrophoresis on Metaphor (BioSpa) gel at 3% or 4% and detected by ethidium bromide staining. Quantification of mutational load was carried out by densitometric analysis using AlphaView software package (Cell Biosciences).

### Alignment of Protein Sequences and Interspecies Conservation Analysis

The interspecies conservation of the amino acidic positions changed by pathogenic mutations and non-synonymous polymorphic variants, detected in our mtDNA complete sequences, was calculated and, as a general rule, all the polymorphisms above a conservation threshold (70%) were considered “possibly synergistic”.

All the available complete mitochondrial protein sequences were downloaded from the non-redundant SwissProt database (http://www.expasy.org/sprot) and three sequence sets were created, corresponding to eukaryotes, vertebrates and mammals. These sequences were aligned as previously reported [58], the most representative amino acidic residues were identified and their prevalence was calculated at each position within the alignment, setting the value of 70% as conservation threshold.

To assess the possible functional effect of primary rare mutations and non-synonymous polymorphic variants four different prediction tools available on line were applied: PolyPhen2, SIFT, SNP&GO and PROVEAN 1.0 [59]–[62]. The accuracy of these tools was tested on the confirmed pathogenic mtDNA mutations in *MT-ND1-6* genes listed in MITOMAP [26]. Eventually, we adopted PolyPhen2 and SIFT that resulted the most accurate algorithms predicting as damaging or not tolerated 15/16 of the tested mutations.

The non-synonymous polymorphic variants were clustered in ancient or recent changes, depending on their position along the phylogenetic tree, and the percentage of conserved and non-conserved amino acid positions was calculated within each cluster. Further analyses of the non-conserved amino acid changes induced by non-synonymous polymorphic variants included the estimate of the local conservation and number of conserved amino acids around the affected position (−10/+10 amino acids), which was compared to the global conservation and the total number of conserved amino acids of the entire protein. Those polymorphisms inducing amino acidic changes at positions with both parameters higher than the global conservation and number of conserved amino acid were considered “possibly synergistic”. Finally, the closest invariant (100% conservation) position for each amino acid change was identified and the polymorphic changes hitting amino acidic positions nearby invariant positions (−4/+4 amino acids) were considered “possibly synergistic”.

## Supporting Information

Figure S1 Pedigrees of LHON families. Family ID numbers, mtDNA mutations and haplogroup affiliations are reported. Symbol definitions are also indicated. Probands are identified by black arrows.
(PDF)

Table S1 Epidemiologic data of LHON diagnosis in the involved centers.
(PDF)

Table S2 Conservation analysis and pathogenicity prediction of mtDNA polymorphic nucleotide changes.
(PDF)

Table S3 Degree of conservation of amino acid positions contiguous to non-conserved polymorphic variants.
(PDF)

Table S4 PCR oligonucleotides and restriction enzymes employed to evaluate heteroplasmy of LHON rare mutations.
(PDF)

Text S1 Case reports: clinical details of patients.
(PDF)
