# Supplementary material for: Rare Primary Mitochondrial DNA Mutations and Probable Synergistic Variants in Leber’s Hereditary Optic Neuropathy
Source: PLoS One. 2012 Aug 3;7(8):e42242. doi: 10.1371/journal.pone.0042242 (PMC3411744; doi:10.1371/journal.pone.0042242)

Figure S1.

Pedigrees of LHON families. Family ID numbers, mtDNA mutations and haplogroup affiliations are reported. Symbol definitions are also indicated. Probands are identified by black arrows.

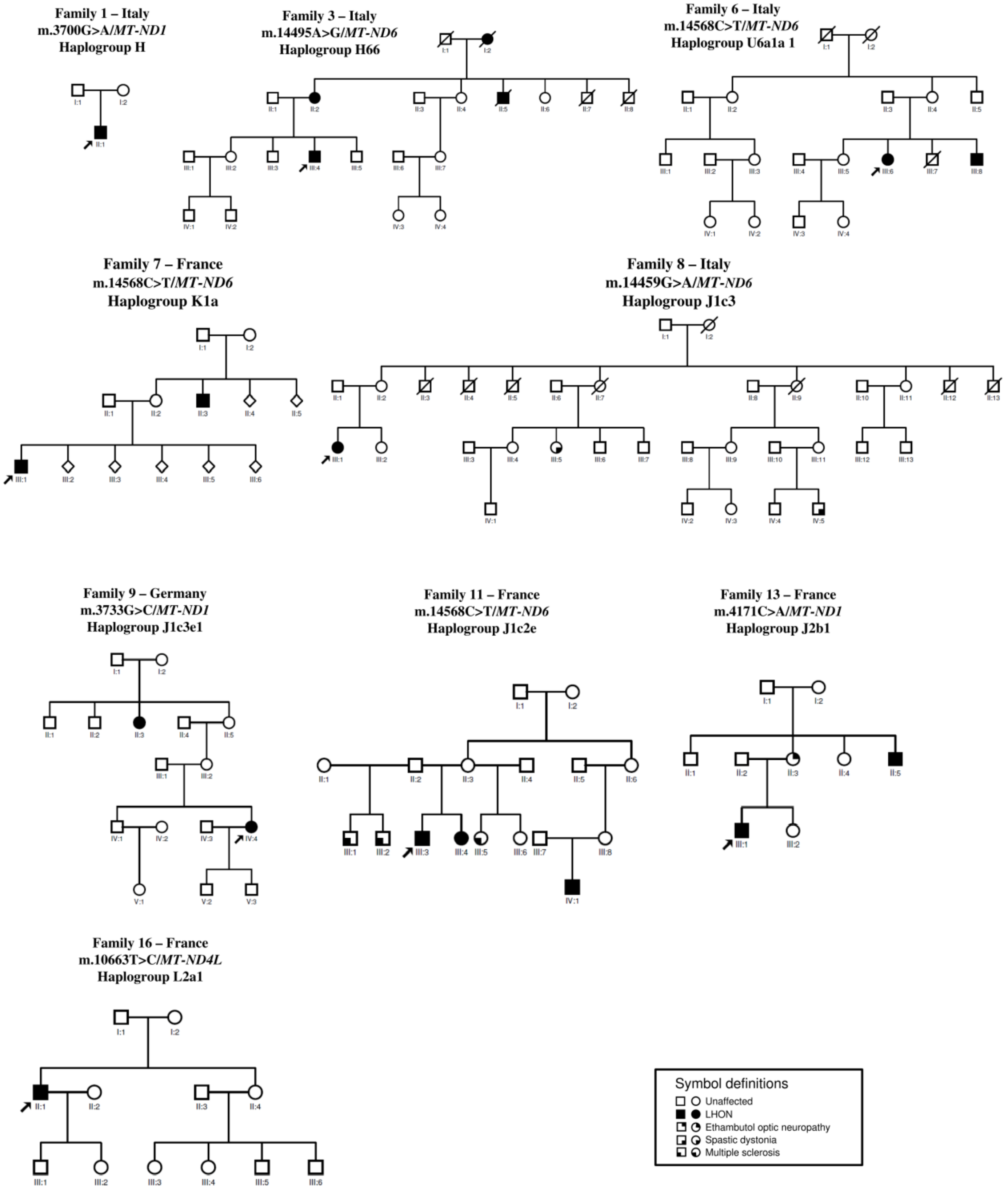

Supplement: Figure S1 — Pedigrees of LHON families. Family ID numbers, mtDNA mutations and haplogroup affiliations are reported. Symbol definitions are also indicated. Probands are identified by black arrows. (PDF) [file pone.0042242.s001.pdf]
