# Supplementary material for: Rare Primary Mitochondrial DNA Mutations and Probable Synergistic Variants in Leber’s Hereditary Optic Neuropathy
Source: PLoS One. 2012 Aug 3;7(8):e42242. doi: 10.1371/journal.pone.0042242 (PMC3411744; doi:10.1371/journal.pone.0042242)
Supplement: Table S1 — Epidemiologic data of LHON diagnosis in the involved centers. (PDF) [file pone.0042242.s002.pdf]

Table S1. Epidemiologic data of LHON diagnosis in the involved centers

	**Italy (2002-2011)**	**France (2001-2011)**	**Germany (1992-2011)**
Optic atrophy diagnosis[a]	371	1085	1140
LHON positive probands	104 (28%)	215 (19.81%)	280 (24.56%)
np11778 (%)	61.5	75.0	73.2
np3460 (%)	17.0	13.0	11.78
np14484 (%)	13.5	9.0	11.07
Number of complete mtDNA sequences	38	100	36
np14568 (%)	1.0	1.4	0.7
np14459 (%)	2.0	-	-
np14495 (%)	1.0	-	-
np14482 (%)	1.0	0.8	0.35
np3700 (%)	1.0	-	0.35
np3733 (%)	2.0	-	0.35
np4171 (%)	-	0.4	-
np10663 (%)	-	0.4	-

[a]Number of index patients tested for LHON mutations (does not include Dominant Optic Atrophy)
