# Supplementary material for: Rare Primary Mitochondrial DNA Mutations and Probable Synergistic Variants in Leber’s Hereditary Optic Neuropathy
Source: PLoS One. 2012 Aug 3;7(8):e42242. doi: 10.1371/journal.pone.0042242 (PMC3411744; doi:10.1371/journal.pone.0042242)
Supplement: Table S2 — Conservation analysis and pathogenicity prediction of mtDNA polymorphic nucleotide changes. (PDF) [file pone.0042242.s003.pdf]

Table S2. Conservation analysis and pathogenicity prediction of the 28 non-synonymous variants identified in the 16 completely sequenced mtDNAs (Figure 1).

**Nucleotide change**	**Gene**	**Amino acid change**	**Eukaryotes Conserv.**	**Vertebrates Conserv.**	**Mammals Conserv.**	**PolyPhen2 Prediction**	**SIFT Prediction**	**Family**
m.4172T>A	*MT-ND1*	p.L289Q	60.39	84.26	100.00	Probably Damaging	Not Tolerated	6
m.4216T>C	*MT-ND1*	p.Y304H	48.05	67.59	62.03	Benign	Tolerated	8, 9, 10, 11, 12, 13
m.5074T>C	*MT-ND2*	p.I202T	48.45	58.73	53.68	Benign	Tolerated	15
m.7299A>G	*MT-COI*	p.M466V	33.57	50.00	56.25	Benign	Tolerated	7
m.7632T>C	*MT-COII*	p.I16T	58.13	86.41	93.21	Possibly Damaging	Tolerated	13
m.7805G>A	*MT-COII*	p.V74I	64.36	83.15	84.57	Benign	Tolerated	6
m.7859G>A	*MT-COII*	p.D92N	51.21	67.93	74.69	Benign	Tolerated	14
m.8393C>T	*MT-ATP8*	p.P10S	42.96	60.64	41.82	Probably Damaging	Tolerated	14
m.8701A>G	*MT-ATP6*	p.T59A	14.80	24.59	36.59	Benign	Tolerated	16
m.9053G>A	*MT-ATP6*	p.S176N	20.40	39.34	58.54	Benign	Tolerated	16
m.9055G>A	*MT-ATP6*	p.A177T	26.00	90.16	90.24	Possibly Damaging	Tolerated	7
m.9091A>G	*MT-ATP6*	p.T189A	50.80	63.93	63.41	Benign	Tolerated	5
m.10398A>G	*MT-ND3*	p.T114A	42.86	61.00	81.08	Benign	Tolerated	7, 8, 9, 10, 11, 12, 13, 15, 16
m.11084A>G	*MT-ND4*	p.T109A	52.48	65.57	86.36	Probably Damaging	Tolerated	14
m.13145A>G	*MT-ND5*	p.S270D	53.77	91.38	92.31	Benign	Tolerated	9
m.13708G>A	*MT-ND5*	p.A458T	31.13	46.55	43.59	Benign	Tolerated	8, 9, 10, 11, 12, 13, 14
m.13759G>A	*MT-ND5*	p.A475T	21.70	41.38	51.28	Benign	Tolerated	1, 7
m.13780A>G	*MT-ND5*	p.I482V	50.00	87.93	82.05	Benign	Tolerated	15
m.13934C>T	*MT-ND5*	p.T533M	23.58	36.21	51.28	Benign	Tolerated	8, 9
m.13966A>G	*MT-ND5*	p.T544A	28.30	36.21	51.28	Benign	Tolerated	14
m.14502T>C	*MT-ND6*	p.I58V	51.61	57.83	76.74	Benign	Tolerated	9
m.14325T>C	*MT-ND6*	p.N117D	21.77	45.78	27.91	Possibly Damaging	Tolerated	12
m.14766C>T	*MT-CYB*	p.I7T	48.43	50.97	57.24	Benign	Tolerated	6, 7, 8, 9, 10, 11, 12, 13, 14, 15, 16
m.14798T>C	*MT-CYB*	p.F18L	68.12	72.67	89.11	Benign	Tolerated	7, 8, 9, 10, 11, 12
m.14927A>G	*MT-CYB*	p.T61A	76.33	81.54	98.13	Benign	Not Tolerated	6
m.15221G>A	*MT-CYB*	p.D159N	42.69	44.49	38.05	Benign	Tolerated	6
m.15257G>A	*MT-CYB*	p.D171N	89.67	93.39	98.21	Benign	Not Tolerated	13
m.15452C>A	*MT-CYB*	p.L236I	42.39	44.24	50.41	Benign	Tolerated	8, 9, 10, 11, 12, 13
m.15812G>A	*MT-CYB*	p.V356M	65.46	68.65	79.59	Benign	Not Tolerated	13
