# Supplementary material for: Rare Primary Mitochondrial DNA Mutations and Probable Synergistic Variants in Leber’s Hereditary Optic Neuropathy
Source: PLoS One. 2012 Aug 3;7(8):e42242. doi: 10.1371/journal.pone.0042242 (PMC3411744; doi:10.1371/journal.pone.0042242)
Supplement: Table S3 — Degree of conservation of amino acid positions contiguous to non-conserved polymorphic variants. (PDF) [file pone.0042242.s004.pdf]

Table S3. Degree of conservation of amino acid positions contiguous to non-conserved polymorphic variants.

**Nucleotide position**[a,b]	**Amino acid position (protein)**	**Interval (-10/+10)**	**Local conservation (%)**	**Global conservation (%)**	**Nearest invariant position (-/+)**
4216	304 (ND1)	294/314	67	77	-6/+11
5074	202 (ND2)	192/212	48	62	-12/+6
7299	466 (COI)	456/476	90	95	-2/+2
**8393**	**10 (ATP8)**	**1/21**	**75**	**65**	**-1/+40**
8701	59 (ATP6)	49/69	73	77	-3/+2
**9053**	**176 (ATP6)**	**166/186**	**77**	**77**	**-1/+22**
9091	189 (ATP6)	179/199	57	77	-14/+10
**13708**	**458 (ND5)**	**448/468**	**68**	**68**	**-1/+7**
13759	475 (ND5)	465/485	48	68	-1/+6
13934	533 (ND5)	523/543	43	68	-6/+2
13966	544 (ND5)	534/554	48	68	-9/+10
14325	117 (ND6)	107/127	14	52	-35/+24
14766	7 (CYTB)	1/21	76	83	-6/+48
**15221**	**159 (CYTB)**	**149/169**	**95**	**83**	**-15/+4**
15452	236 (CYTB)	226/246	57	83	-72/+48

[a] Nucleotide changes affecting amino acid residues with a local conservation equal or higher than the global conservation are in bold.

[b] Nucleotide changes affecting amino acid residues close to invariant amino acids (+4/-4 aa) are underlined.
