# Supplementary material for: Rare Primary Mitochondrial DNA Mutations and Probable Synergistic Variants in Leber’s Hereditary Optic Neuropathy
Source: PLoS One. 2012 Aug 3;7(8):e42242. doi: 10.1371/journal.pone.0042242 (PMC3411744; doi:10.1371/journal.pone.0042242)
Supplement: Table S4 — PCR oligonucleotides and restriction enzymes employed to evaluate heteroplasmy of LHON rare mutations. (PDF) [file pone.0042242.s005.pdf]

Table S4. PCR oligonucleotides and restriction enzymes employed to evaluate heteroplasmy of LHON rare mutations [a,b].

**Map Locus-Mutation**	**Primer**	**Nucleotide position**	**Sequence**	**Restriction Enzyme**
m.3700G>A/*MT-ND1*	Fw	3441-3460	ACTACAACCCTTCGCTGACG	*HhaI*
	Rv	3940-3920	TGAAGCCTGAGACTAGTTCGG	
m.3733G>C/*MT-ND1*	Fw	3501-3520	ATCTACCATCACCCTCTACA	*NdeI*
	Rv	3940-3920	TGAAGCCTGAGACTAGTTCGG	
m.4171C>A/*MT-ND1*	Fw	4011-4030	CACCCTCACCACTACAATCT	*NdeI*
	Rv	4301-4281	TTACTCTATCAAAGTAACTCT	
m.10663T>C/*MT-ND4L*	Fw	10357-10377	TAAGTCTGGCCTATGAGTGA	*BcuI*
	Rv	10930-10911	GGAAAAGGTTGGGGAACAGC	
m.14459G>A/*MT-ND6*	Fw	14430-14458	ATGCCTCAGGATACTCCTCAATAGCCGTC	*MaeIII*
	Rv	14594-14563	ATGGGGGTTTAGTATTGATTGTTAGCGGTG	
m.14495A>G/*MT-ND6*	Fw	14464 -14494	AGTATATCCAAAGACAACCATCATTCCCCAT	*NlaIII*
	Rv	14594-14563	ATGGGGGTTTAGTATTGATTGTTAGCGGTG	
m.14568C>T/*MT-ND6*	Fw	14407-14424	CAAGACCTCAACCCCTGA	*AciI*
	Rv	14705-14682	CATTGGTCGTGGTTGTAGTCCGTG	

[a] The mutations 3733G>A/*MT-ND1* and 14482C>A/*MT-ND6* were already tested by Valentino et al. 2004[8] and Valentino et al. 2002 [11], respectively.

[b] In addition the possible synergistic mutation 4172T>A/*MT-ND1* was tested by using the enzyme *BseMI* and the primers Fw/4151-4171(ACGACCAACTCATACACCTGC) and Rv/4301-4281 (TTACTCTATCAAAGTAACTCT).
