# Supplementary material for: Rare Primary Mitochondrial DNA Mutations and Probable Synergistic Variants in Leber’s Hereditary Optic Neuropathy
Source: PLoS One. 2012 Aug 3;7(8):e42242. doi: 10.1371/journal.pone.0042242 (PMC3411744; doi:10.1371/journal.pone.0042242)
Supplement: Text S1 — Case reports: clinical details of patients. (PDF) [file pone.0042242.s006.pdf]

Text S1.

Case reports: clinical details of patients.

All analyzed patients were highly suspected to suffer from LHON based on typical clinical features and evidence of maternally inherited disease in those cases with multiple affected individuals (Figure 3). All were negative for the three common LHON primary mutations. For the seven previously reported cases (Families 2, 4, 5, 10, 12, 14 and 15 in Table 1) we refer to published studies.[1-6] We here add the clinical reports of the remaining nine unpublished families (10 patients) included in this study.

Family 1 – individual II:1

The proband from the Italian family 1 is a 28-year-old male who experienced a bilateral visual loss. The first ophthalmologic exam, thirteen days after onset, showed hyperemic optic nerve head with microangiopathy, central scotoma and the visual acuity was counting fingers in OD and 1/10 in OS. The neurologic exam performed a few days later was unremarkable, except for brisk deep tendon reflexes and a mild postural tremor at the upper limbs. Brain CT scan and MRI, and cerebrospinal fluid were all normal. Pattern visual evoked potentials (P-VEPs) showed the absence of cortical responses in OS, whereas in OD they had reduced amplitude and prolonged latencies. Visual acuity at this time was 1/100 in both eyes. The patient was treated with corticosteroids and a slight subjective improvement of visual acuity was reported. We examined this patient four months after disease onset and his visual acuity was 1/20 in OD and 1/10 in OS. The fundus examination showed temporal atrophy with preservation of nerve fiber layer in the other sectors, as also documented by the optical coherence tomography (OCT) exam.

Family 3 – individual III:4

The 43-year-old proband from the Italian family 3 complained of poor vision in OD when he was 22, after a mild trauma in OS. He was a heavy smoker (3 packs of cigarettes a day) since he was 13. Retrobulbar corticosteroid administration was ineffective. After about 20 days, visual loss occurred also in OS and a therapy with high dosage of vitamin B12 was started. Within a year he progressively improved his vision and fluctuations of visual acuity were reported in the following years. He also suffered frequent episodes of migraine with aura (up to four per month).

The family history was remarkable for his mother who lost vision bilaterally at 29 years of age during a breast-feeding period after her second delivery, his maternal uncle who lost vision at 22 years of age, and his maternal grandmother who was reported with poor vision since her thirties.

Family 6 – individuals III:6 and III:8

The first patient (III:6) is a 39-year-old Italian woman who experienced at age 13, subacute and painless loss of central vision bilaterally, accompanied by photophobia and green/blue

photopsias. The ophthalmological examination documented a central scotoma. Administration of corticosteroids for two weeks was ineffective. The patient had a partial recovery of visual acuity during the subsequent six months. At the time of our observation she complained of photophobia, emeralopia, muscular fatigue and transient blurring of vision after intense physical exercise. Neurological examination was unremarkable. Serum lactic acid was slightly elevated after standardized exercise (27.3 mg/dl, normal range 5.8-22 mg/dl) and pyruvic acid was also slightly increased (1.09 mg/dl, normal range 0.36-0.83). Skeletal muscle biopsy presented non-specific myopathic changes. P-VEPs showed reduced amplitude and markedly increased latencies of cortical responses bilaterally with normal electroretinogram. At ophthalmological exam visual acuity was 4/10 in OD and 3/10 in OS. Visual fields showed bilateral central scotoma and OCT documented a generalized reduction of retinal nerve fibers, more evident on the temporal quadrant. Brain CT scan and EKG were unremarkable.

The 34-year-old brother of the proband (III:8), complained loss of visual acuity in OS (6/10) at 12 years of age and at 17 years, after a trauma in the left eye while playing soccer, he presented a bilateral reduction of visual acuity (6/10 in both eyes at ophthalmologic examination) with pale optic discs and central scotoma. The patient worsened over the subsequent year, and then slowly recovered visual acuity. At our observation his neurological examination was unremarkable. Serum lactic acid after standardized exercise was normal and pyruvic acid was slightly elevated (0.99 mg/dl, normal range 0.36-0.83). Skeletal muscle biopsy showed non-specific myopathic changes. P-VEPs showed reduced amplitude and increased latency of cortical responses bilaterally, more evident in OS, with normal electroretinogram. The ophthalmological exam showed a visual acuity of 8/10 in OD and 5/10 in OS and generalized reduction of the nerve fiber layer thickness at OCT, more pronounced in the temporal quadrant. Brain CT scan and EKG were normal.

Family 7 – individual III:1

The proband is a 20-year-old French male of Lebanese ancestry who presented an acute visual loss in OS at the age of 18 years. Fundus examination showed a swollen optic disc bilaterally. Visual acuity was counting fingers in OS and 9/10 in OD. Color vision was abnormal at the Ishihara test in OD and untestable in OS. Visual field examination disclosed a bilateral central scotoma and at cerebral MRI both optic nerves had increased signals in the retrobulbar portion, especially on the right side. After one month, visual acuity was further decreased in OD (1.5/10) and counting fingers in OS. Six months after onset, the patient’s visual acuity was counting fingers at 20cm bilaterally with central scotoma and optic disc pallor in both eyes. Family history was remarkable for a maternal uncle who presented bilateral optic atrophy.

Family 8 – individual III:1

This 31-year-old Italian woman suffered a rapidly sequential (within days), painless visual loss in both eyes, more severe in OS, when she was 27. She also complained of impaired color vision and photophobia. Corticosteroid therapy did not improve her vision. Three months after onset her neurological examination was normal except for diffusely brisk deep tendon reflexes. Serum lactic acid after exercise was bordeline (22.9 mg/dl, normal range 5.8-22 mg/dl). At ophthalmological examination her visual acuity was counting fingers in both eyes with bilateral central scotomas. She was treated with idebenone (270 mg/day) and six months after disease onset she started to recover vision, reaching 1.25/10 bilaterally at one year after clinical onset with a consistent reduction of the scotoma. At last follow-up there were no further changes in visual acuity and a generalized atrophy of the optic nerve was evident. The family history was remarkable for the presence of two distantly related maternal relatives, who were described as wheelchair bound and suffering of an unspecified form of "paralysis", but with normal vision.

Family 9 – individual IV:4

The proband, a 48-year-old woman, suffered secondary glaucoma in OS due to a malignant melanoma of the ciliary body, for which she underwent surgical resection. Four weeks later, she presented progressive loss of visual acuity in OD, which worsened in the next six months. She was treated with high dose methylprednisolone without benefit. Two years later the best-corrected visual acuity in OD was hand motion and 1/50 in OS. Visual field documented a large central scotoma bilaterally with a small peripheral sparing in OS. Intraorbital and cerebral MRI showed a minimal enhancement of the right optic nerve. In the following three years, visual acuity stabilized, without spontaneous improvement. Family history was remarkable for a sister of the proband's maternal grandmother, who suffered an unexplained blindness in her middle age.

Family 11 – individual III-3

This 68-year-old French patient had a late onset loss of vision when he was 62 in OD followed by OS four months later. At this time visual acuity was 1/80 OD and 1/40 OS with bilateral central scotoma. Ophthalmologic examination revealed telangectatic blood vessels at the optic discs. Idebenone treatment was started (270 mg/day) and administered for 30 months. The patient experienced a further slow decrease of visual acuity, reaching counting fingers bilaterally with optic disc pallor at fundus examination. Brain MRI showed non-specific hyperintense changes of the cerebral white matter. Two years after onset, the patient experienced a slow improvement of his visual acuity (1/50 OU).

His family history was remarkable for one sister who suffered visual loss and two brothers and one sister who suffered multiple sclerosis. All three died before the age of 50 years. Furthermore, a young son of a maternal cousin experienced subacute loss of visual acuity reaching

1/20 bilaterally when he was 19. His neurological examination and cerebral MRI were unremarkable.

Family 13 – individual III:1

This 23-year-old French male with an Algerian father and a French mother suffered severe acute loss of vision in OD (1/10) when he was 16. Three weeks later he lost vision in OS (5/10). At fundus examination the optic discs were pale with telangectatic vessels and visual fields showed large central scotomas. P-VEPs showed the absence of cortical responses bilaterally and cerebral MRI demonstrated thinning of the right optic nerve with hyperintense signal in both optic nerves and chiasm. Neurological examination was unremarkable. At 17 years his visual acuity was counting fingers at 1 m in both eyes. At this time a treatment with coenzyme Q10 (180 mg/day) was started in association with vitamins B. At age 19, his visual acuity was 5/10 in OD and 4/10 in OS and two years later his visual acuity was 1/10 in OD and further improved to 8/10 in OS. OCT confirmed a significant reduction of retinal nerve fiber layer thickness. A new MRI showed a severe optic atrophy with hyperintense signal in T2-weighted sequences without other brain lesion. Remarkably, the proband's mother suffered toxic optic neuropathy due to ethambutol treatment for pulmonary tuberculosis reversed after therapy withdrawal. Furthermore, also a maternal uncle suffered visual loss.

Family 16 – individual II:1

This 60 year-old French man, originally from Benin, complained at 17 years of age a rapid loss of visual acuity in both eyes. His best corrected visual acuity at last ophthalmologic examination was less than 4/10 bilaterally. Fundus examination showed bilateral optic atrophy, little drusen-like intraretinal deposits, following the temporal arcades trajectory and surrounding optic disk.
